# Three-week exercise and protein intake immediately after exercise increases the 6-min walking distance with simultaneously improved plasma volume in patients with chronic cerebrovascular disease: a preliminary prospective study

**DOI:** 10.1186/s13102-022-00429-x

**Published:** 2022-03-15

**Authors:** Chika Sato, Yoshi-ichiro Kamijo, Yuta Sakurai, Shohei Araki, Yuki Sakata, Ayana Ishigame, Kota Murai, Izumi Yoshioka, Fumihiro Tajima

**Affiliations:** 1 Nachi-Katsuura Research Centre of Sports Medicine and Balneology, Nachi-Katsuura Balneologic Town Hospital, 1185-4 Tenma-Nachi-katuurachou, Higashimuro gun, 649-5331 Japan; 2 Department of Rehabilitation Medicine, School of Medicine, Wakayama Medical University, 811-1, Kimiidera, Wakayama, 641-8509 Japan; 3 Institute of Sports Science and Environmental Physiology, Medical Centre for Health Promotion and Sports Science, Wakayama Medical University, 2-1 Honmachi, Wakayama, 640-8033 Japan; 4 Department of Rehabilitation Medicine, Dokkyo Medical University Saitama Medical Center, 2-1-50 Minami-Koshigaya, Koshigaya, Saitama 343-8555 Japan

**Keywords:** Blood volume, Physical endurance, Cerebrovascular disorders, Dietary supplements, PROr

## Abstract

**Background:**

Blood volume (BV) is a critical factor for physical endurance in chronic stroke patients, while hypervolemia can worsen hypertension in these patients. This prospective study assessed whether rehabilitation combined with protein supplementation immediately after each exercise for 3 weeks would improve plasma volume (PV) and BV as well as physical endurance without worsening hypertension.

**Methods:**

Ambulatory patients with chronic cerebrovascular disease who received a 3-week rehabilitation program with high protein jelly (intervention group [PG]; n = 8; 10-g protein) or protein-free jelly (control group [CG]; n = 8) consumed within 30 min after each exercise. PV and BV were assessed while measuring the 6-min walking distance (6MWD), peak oxygen consumption (VO_2peak_), strength of knee extension, and resting blood pressure before and after the intervention. Two-way ANOVA was used to determine whether there was an interaction of time × group. The difference between before and after intervention or between the groups by post-hoc test (Tukey’s test) at the level of *P* < 0.05.

**Results:**

The 6MWD increased only in the PG (*P* = 0.001; an interaction of Group and Time, *P* = 0.037). PV and BV increased only in the PG (*P* < 0.05). VO_2peak_ and strength of knee extension in the paralysed limb increased in both groups (*P* < 0.05). The resting blood pressure did not worsen after the intervention.

**Conclusions:**

In chronic post-stroke patients, 3-week rehabilitation combined with protein intake immediately after exercise increased 6MWD simultaneously with increased PV and BV, but it did not increase resting blood pressure. The present regimen is acceptable and effective for ambulatory patients with chronic cerebrovascular disease.

*Name of the registry* Examining effects of protein supplementation on functional improvement during rehabilitation intervention in chronic stroke patients

*Trial registration number* UMIN000028009; date of registration: 30/06/2017. This study was registered prospectively.

**Supplementary Information:**

The online version contains supplementary material available at 10.1186/s13102-022-00429-x.

## Introduction

The number of patients with cerebrovascular disease is approximately 1.1 million in Japan [1]. Approximately 850,000 patients have received long-term nursing care through government support [2, 3]. Physical function, especially independent walking, plays a key role in improving the quality of life of patients (e.g. continuing to live at home) and reducing medical costs under the Medicare system [4].

Physical function is generally associated with physical endurance and muscle strength of the extremities. In a clinical rehabilitation setting, the 6-min walking distance (6MWD) has been used to assess physical endurance and muscle strength of the extremities also relates it [5]. Patients are reported to walk at a self-selected speed as fast as possible within 6 min. The main outcome was 6MWD; intra-class correlation coefficients < 10% in patients with low physical endurance, and peak oxygen consumption rate (VO_2peak_) between 14 and 17 mL/kg/min [6]. The 6MWD was applied to patients with chronic cerebrovascular disease [6]. A 30-min treadmill walking intervention performed 3 days per week for 4 weeks increased 6MWD by 11%, with increased muscle strength on both the non-paretic and paretic sides [7]. High-intensity treadmill training that included 3 sessions per week, with each session between 50 and 60 min, increased 6MWD by 20% after 3 months and increased step length of paretic side by 2 cm [8]. These previous studies suggest that improvement in 6MWD can be achieved through rehabilitation for a duration of at least 4 weeks and is associated with increased muscle strength of the non-paretic and/or paretic lower limb. However, it remains unknown whether other mechanisms, such as physical endurance, can result in improved 6MWD in patients with chronic cerebrovascular disease.

VO_2peak_ is one of the indexes for physical endurance, which must be ≥ 14 mL/kg/min to maintain a walking velocity of ≥ 0.9 m/s [9], and is correlated with 6MWD [10–12]. In contrast, VO_2peak_ was positively correlated with plasma volume (PV) and/or blood volume (BV) [13]. Therefore, rehabilitation may improve 6MWD simultaneously with increased PV or BV, even in patients with chronic cerebrovascular disease. Recently, Araki et al. (*in submission*) showed that VO_2peak_ in patients with chronic cerebrovascular disease was 14.5–30.0 mL/kg/min and tended to correlate with BV. PV and/or BV may be critical factors for physical endurance, even in the population.

However, PV or BV expansion is associated with increased blood pressure in elderly and/or hypertensive subjects [14], which are risk factors for cerebrovascular diseases [15]. As previously shown, in healthy elderly participants, a regimen of combined exercise and ingestion of both carbohydrates and proteins immediately after each exercise improved PV and BV [16]. Volume expansion was observed in hypertensive patients after an 8-week aerobic exercise intervention combined with intake of glucose and protein; however, the regimen did not cause increased blood pressure [14]. The previous results suggest that the regimen may increase PV and/or BV without increasing blood pressure, even in hypertensive subjects.

This study aimed to test the hypothesis that a combination of rehabilitation therapy plus protein intake immediately after each exercise session increases PV, BV, and 6MWD in patients with chronic cerebrovascular disease. Furthermore, we assessed that even if BV was increased in patients after the intervention, blood pressure would not increase, as previously shown in elderly hypertensive individuals [14].

## Methods

### Participants

**Fig. 1:**
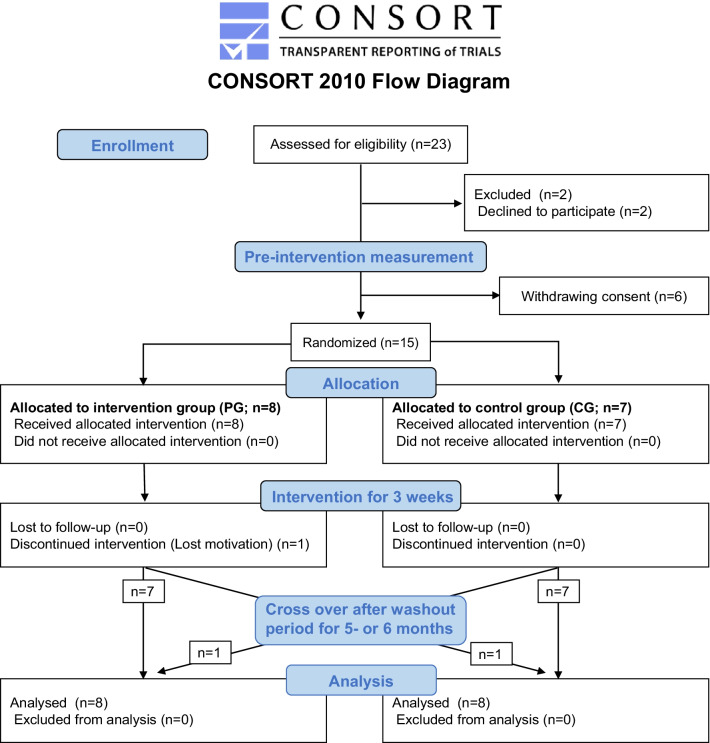
CONSORT Flow Diagram. We recruited 23 patients, who were admitted to the Nachi-Katsuura Balneologic Town Hospital between June 2017 and December 2018. Two patients refused their participation. Six patients withdrew their consent after the pre-intervention measurements. Fifteen patients were allocated into the intervention (PG; n = 8) and the control groups (CG; n = 7). One patient in the intervention group discontinued intervention due to lost motivation. Each one of both groups performed both trials, after a wash-out period of 5 or 6 months. Finally, 8 patients in each group completed this trial

This study was conducted in accordance with the Declaration of Helsinki, and the protocol was approved by the ethics review board of Wakayama Medical University (#2049). All participants signed a consent form voluntarily before participating in the study. The right to withdraw consent at any time without stating the reason was guaranteed without any individual disadvantage for subsequent medical care. The inclusion criteria in the present study were patients who had chronic cerebrovascular disease for > 6 months, walked independently regardless of using foot orthosis or walking aids, and had normal cognitive function (Hasegawa dementia rating scale-revised score > 20 [17]). All patients could communicate effectively and understood the study objectives. Even patients who used β-blockers were included if the resting heart rate was not low and increased over the target heart rate during exercise. We recruited 23 patients who were admitted to the Nachi-Katsuura Balneologic Town Hospital between June 2017 and December 2018. Two patients refused to participate. Six patients withdrew consent after the pre-intervention measurements. Each patient provided signed consent before participating in the study. Exclusion criteria were patients with brainstem stroke, bone and joint problems, heart or respiratory diseases that could worsen with walking, liver, or kidney diseases, poorly controlled diabetes, nephropathy stage 3 or higher, and/or drug allergy. Four patients had mild anaemia and one patient had moderate anaemia [18]; however, all 5 patients were included in the study. None of the patients participated in any rehabilitation therapy in the 2-month period before the study, except for one patient in the intervention group (PG; protein was ingested just after each rehabilitation) who underwent 20-min of rehabilitation therapy at an outpatient clinic at our hospital twice a week for 9 years. Fifteen patients were allocated to the PG (n = 8) and control group (CG; n = 7). One patient in the intervention group discontinued the intervention due to lack of motivation. One patient of each group performed both trials after a wash-out period of 5 or 6 months. Finally, eight patients in each group completed the trial. (Fig. 1).

Assuming that the main effects of d (=|μ_1_ − μ_2_|/SD) (d, effect size; μ_1_ and μ_2_, two independent means) for PV were 1.75 (44.6 (6.7) and 47.5 (6.3) mL/kg, mean (SD), before and after the intervention in hypertensive elderly participants who ingested protein just after each exercise [14]), the correlation before and after intervention was > 0.96, the statistical power (1 − β) was > 0.95 at an α of 0.05, and the required sample size was 7 for the paired t-test (G*Power 3.1, Kiel, Germany).

### Experimental design and intervention

This was a preliminary prospective intervention, and a double-blind study involving one patient from each group who performed both trials, after a wash-out period of 5 or 6 months. Every two patients were admitted to the hospital. Participants were randomly assigned to two groups using the permuted block method (block size 2): protein ingestion immediately after each exercise session (PG; the intervention group; n = 8) or ingestion of protein-free jelly (CG; n = 8) by a study evaluator who worked at another hospital. The doctor who was working in the hospital (C.S.) enrolled participants but was not informed which groups they belonged to. Patients in the PG received a protein-rich jelly (RehaTime Jelly; Clinico Co.; Tokyo; 100 kcal, 10 g protein, 15 g carbohydrate, 0 g fat, and 4 mg sodium in 120 g); meanwhile, those in the CG were protein-free (Minute-Made Qoo; Coca-Cola Japan Co.; Tokyo; 90 kcal, 0 g protein, 18 g carbohydrate, 0 g fat, and 4–16 mg sodium in 125 g). Each participant was handed in a cup with jelly by a nurse who did not participate in the study. Patients ingested one pack of jelly within 30 min of the exercise session. The idea of protein ingestion immediately after each exercise session was based on the results of a previous study [16]. None of the patients, care providers, and investigators were informed about which jelly was administered until the end of the study. The two patients who received both supplements did not notice any difference in flavour between the two supplements.

### Rehabilitation program

**Fig. 2:**
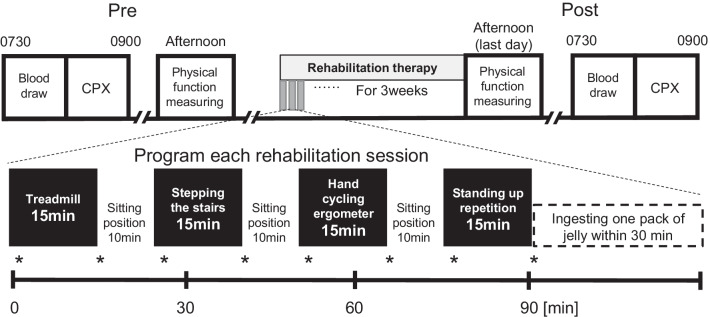
Study protocol. Each patient received in-hospital 11-h rehabilitation program per week for 3 weeks. Each rehabilitation session lasted 1 h, the 1st one was conducted in the morning and another in the afternoon on 5 weekdays, and one session was done on Saturday morning. Treadmill; walking on treadmill (WEBG3300, Reha-Tread G, Senoh Corporation, Chiba, Japan) at a speed adjusted according to each patient. Stepping the stairs; climbing up and down the stairs in the hospital. Hand cycling ergometer; cycling upper body ergometer (WBK284740H, Rehab Trainer 881E, Monark Exercise AB, Sverige, Sweden) with their hands at wattage adjusted according to each patient. Standing up repetition; standing up from a chair, sitting down and repeating. The Borg’s scale of each patient’s program was recorded. Patients of the intervention group received protein-rich jelly while those of the control group received protein-free jelly within 30 min after the rehabilitation session. Physical fitness tests were performed in the evening of the day before starting the rehabilitation program and the last day of 3-week program. Blood samples were drawn and plasma volume was measured during 7:30–8:00 am before breakfast on the same days as above. *, blood pressure and pulse rate were checked. CPX, Cardiopulmonary exercise testing.

**Fig. 3:**
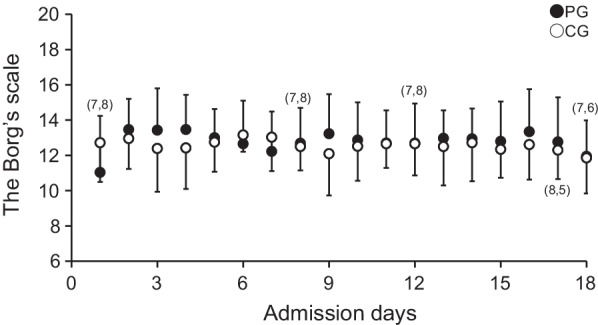
The Borg’s scale during intervention in the Black dot, PG; White dot, CG. To determine differences in the Borg’s scale during intervention between groups, unpaired t-test was applied at each time point during day 1 to 18. Because of lacks of data at day 1, 8, 12, 17 and 18, numbers of data at these days are shown in parentheses. Differences were considered statistically significant at *P* < 0.05. Statistical evaluations were performed using SPSS (version 24.0; IBM, Chicago, IL). Values are represented as mean (SD) unless otherwise stated. All participants performed rehabilitation for 33 sessions

All patients in the study underwent 11 sessions of rehabilitation program per week for 3 weeks. Each rehabilitation session lasted for one hour. The 1st session was conducted in the morning and the other in the afternoon on 5 weekdays per week, and only one session was conducted on Saturday morning per week. The total number of training days was 18 and 33, respectively. The rehabilitation program consisted of standing/sitting exercises, aerobic exercise with a hand cycling ergometer, gait training on a treadmill, and stepping-stairs training (Fig. 2). We attempted to establish an exercise intensity with different parameters, such as the velocity of a treadmill walk, workload of an ergometer, and number of repetitions for standing/sitting; therefore, Borg’s scale was 11–13 (Fig. 3).

### Meals

The patients’ diet was provided during admission with the following composition (values per day): 23.66–35.06 kcal/kg of body mass (BM), carbohydrates 3.23–4.89 g/kg of BM, proteins 1.11–1.38 g/kg of BM, lipids 0.72–1.01 g/kg of BM and salt 0.11–0.14 g/kg of BM.

### Protocol for measurements

On the 1st day of admission, each patient reported to the laboratory at 7.30 am normally hydrated but in a fasting state for at least 9 h before the measurement. After emptying their bladder, the patient was weighed and asked to take the supine position throughout the measurement in a temperature-controlled room at 25–28 °C. A 21-gauge butterfly needle was inserted into the antecubital vein for blood sampling and dye injection to assess the PV and baseline values of blood constituents. After a 30-min rest, 14 mL and 12 mL of blood samples were obtained for baseline values before and after the intervention, respectively, followed by injection of sterilised Evans blue dye [19]. Then, 3 mL of blood sample was collected 10 min after the injection. Whole blood samples were used to determine haematocrit (Hct) and haemoglobin concentration ([Hb]). Plasma and serum were separated after centrifugation and were used to determine the PV, plasma hormone concentration, and general biochemistry.

A cardiopulmonary exercise test was performed following PV measurement using a graded exercise method [5, 20]. Before this test, blood pressure was measured once in a seated position after a 60-min rest following PV measurement. Then, the patient sat and rested on an ergometer while all the equipment was attached. After 2-min of rest measurements, the patient started pedalling at 50 revolutions/min without loading. Then, the workload was increased by 5 W every 3 min until it reached 15 W and above this intensity, 5 W every one min for female patients. Meanwhile, for male patients, the workload was increased by 10 W every two min until it reached 30 W, and above this intensity, 15 W every one min. This procedure was performed until male and female patients could not maintain the rhythm due to exhaustion. We measured VO_2_ every 15 s (MetaMax 3B, Cortex, Leipzig, Germany) and monitored it continuously at rest and during the graded exercise to record heart rate every minute. The same procedure was performed one or two days before discharge at the same time of the day.

Physical fitness tests were performed in the evening of the day before and on the last day of the 3-week program. We measured the 6MWD for each patient and 10-m walking speed at optimum and maximum (10MWS) level to calculate the walking velocity. The patients were allowed to use usual foot orthoses or walking aids if required. The strength of the quadriceps femoris muscle was measured in a seated position. A skilled physiotherapist scored the Fugl-Meyer Assessment (FMA) to estimate the severity of the disability of each patient before the onset of the intervention. The same measurements were performed one day before discharge at the same time of the day.

### Measurements

The primary outcome was change in PV before and after the intervention. Also, we assessed changes in BV, plasma concentrations of stress-related hormones or catecholamines, VO_2peak_ and 6MWD at optimal speed, 10MWS at optimal and maximal efforts, and strength of knee extension and resting blood pressure.

#### PV

Absorbance (620 and 740 nm, SH-1000 Lab; Corona Electric, Hitachinaka, Japan) of plasma at baseline and 10 min after dye injection was measured. PV was calculated as follows [19, 21]:

$${\text{PV}} = {\text{ }}\frac{{{\text{EB }} \times {\text{ D }} \times {\text{ OD}}_{{{\text{stand}}}} }}{{({\text{OD}}_{{10\,\min }} - OD_{{{\text{Blank}}}} ) \times 1.03}}$$

where EB is the volume (mL) of the dye injected; D is the dilution of the standard dissolved in distilled water (× 1000 in the present study); OD_stand_ is the absorbance of the standard at 620 nm; OD_Blank_ and OD_10min_ are the absorbances of the plasma sampled before the dye injection and at 10 min after injection, respectively; and 1.03 is a factor introduced to correct for slow dye uptake by the tissues [19]. The background absorbance due to turbidity was corrected using a regression equation for the relationship between 620 and 740 nm in the present study [5].

#### BV

BV was calculated using the following equation: BV = PV/(1 − Hct/ 100) [13]. These values were divided by body weight.

#### VO_2peak_ and peak HR

VO_2peak_ were determined by averaging the three largest consecutive values at the end of the exercise. Peak HR was applied as the value at the last minute of the VO_2peak_ test.

#### 6MWD

Participants performed a 6-min walking test based on a standardised protocol [22]. The walking course was 30 m in length, and patients were instructed to walk back and forth on the course as fast as possible for 6 min. Each instruction and encouragement was verbalised simultaneously based on the standardised protocol.

#### 10MWS

10-m walking speed at optimum and maximum speed were tested in triplicate. The peak values at each speed were obtained to calculate walking velocity. The course of the 10MWS consisted of a 2 m warm-up, 10 m used for the speed measurement, and 2 m for slowing down to a stop, a total of 14 m. Patients were instructed to walk at a self-selected comfortable pace at the optimum speed and as fast as possible at maximum speed, while measuring 10-m time with a stopwatch [23, 24] to two decimal places.

#### Knee extension strength

**Fig. 4:**
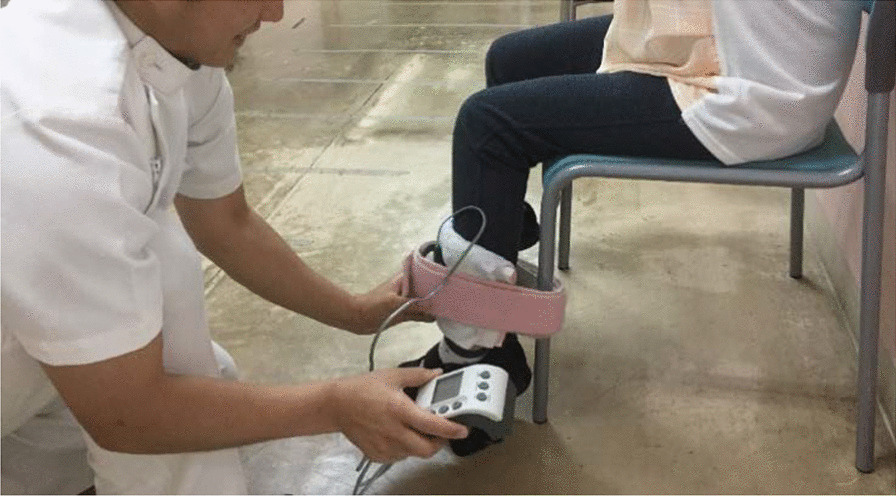
Measurement of knee extension strength with hand-held dynamometer. The patients sat on a chair and their hips and knees flexed 90 degrees [25–27]. An examiner put the dynamometer at 20 cm distal to their knee joint. Patients were reported to extend the knee joint as possible as they could

The strength of the quadriceps femoris muscle was measured with a hand-held dynamometer (Power track II MMT commander, MF-104AA, Nihon Medix, Chiba, Japan) in duplicate, and the peak value was obtained. Power track II MMT commander is a battery-operated hand-held device, which measures the peak force in Newtons (N) up to approximately 600 N (125 lb). The patients sat on a chair with their hips and knees flexed at 90° [25–27]. An examiner placed the dynamometer 20 cm distal to their knee joint. Patients were instructed to extend the knee joint as much as possible (Fig. 4).

#### FMA

A skilled physiotherapist scored this test to confirm that the patients’ impairments were comparable between the groups. The Fugl-Meyer Assessment (FMA) is a stroke-specific, performance-based impairment index [28]. This index consists of assessments of motor function of the upper and lower limbs, sensory function, balance, ranges of motion, and joint pain during physical activity of each sub-score was 66, 34, 24, 14, 44, and 44, respectively; the total score was 226.

#### Cardiovascular responses

Systolic (SBP) and diastolic blood pressures (DBP) were measured using an automatic equipment (UA-787, A&D, Tokyo) once patients were in a seated position after a 60-min rest after completion of PV measurement before and after the intervention period. We reported the blood pressure before the cardiopulmonary exercise test as resting blood pressure (shown in Table 5), while SBP and DBP were also measured during a rehabilitation session before and immediately after each exercise. Blood pressure was remeasured only when the previous measurement was erroneous. The coefficient of variation of the measurement was 4.6–7.1% of SBP, 3.4–6.3% of DBP, and 2.1–3.8% of pulse rate in four healthy or hypertensive adults in our department. The pulse pressure was calculated as SBP–DBP. The mean arterial pressure was calculated as DBP + (SBP − DBP)/3. The rate-pressure product was calculated as the heart rate × SBP.

#### Blood analyses

A 1-mL aliquot of 14 mL or 12 mL was used to determine Hct (in %, microcentrifuge; the coefficient of variation was 0.69%) and [Hb] (in g/dL, sodium lauryl sulphate haemoglobin method; Sigma Chemical, St. Louis, MO; < 1.5%) in triplicate. Six of 14 mL (before the intervention) or 12 mL (after the intervention), which were obtained at baseline, and 3 mL of blood obtained 10 min after injection were transferred into a heparin-treated tube and centrifuged at 4 °C at 3000 rpm for 30 min. Approximately 1 mL of separated plasmas were used to determine the PV. The remaining plasma at baseline was used to determine cortisol, catecholamine, and aldosterone concentrations. Seven of 14 mL and five of 12 mL of the blood samples at baseline before and after the intervention, respectively, were transferred into a serum separator tube and centrifuged at 24–25 °C for 20 min. The remaining serum obtained before the intervention was used to assess general biochemistry (TBA-120FR, TOSHIBA, Tochigi, Japan) and HbA1c (HLC-723G11, Tosoh, Tokyo). Analysis of cortisol, catecholamine, and aldosterone concentrations in plasma was performed at an external testing laboratory (SRL, Hachioji, Japan). The plasma and serum obtained after PV measurement were stored at − 80 °C until the next assay. Plasma adrenaline and noradrenaline concentrations were measured using high-performance liquid chromatography. The respective intra-assay coefficients of variation (CV) for adrenaline, noradrenaline, and dopamine were 5.04%, 3.59%, and 5.71% at the levels of 255, 248, and 257 pg/mL, respectively. Plasma cortisol concentration was measured using electro chemiluminescence immunoassay, with 4.31% of the intra-assay CV at 11.58 μg/dL. Plasma aldosterone concentration was measured by chemiluminescent enzyme Immunoassay, with the intra-assay CV 3.47% at 66.22 pg/mL.

### Statistical analysis

The χ^2^ test was used to determine the sex and type of disease bias, haemorrhage, or infarction. A normal distribution was assumed. Two-way ANOVA (within factor, time; between factor, group) was used to determine whether there was an interaction of time × group. The difference between before and after intervention or between the groups (PG and CG) was determined by post-hoc test (Tukey). To determine differences in the Borg’s scale during intervention between groups, an unpaired t-test was applied at each time point from day 1 to 18, since several data points were lacking, specifically on days 1, 8, 12, 17, and 18. Differences were considered statistically significant at *P* < 0.05. All statistical evaluations were performed using SPSS (version 24.0; IBM, Chicago, IL). Values are represented as the mean (SD) unless otherwise stated.

## Results

Some examples of potential adverse events in our study include the following: allergy to jelly we provided or to Evans blue dye, injury or onset of joint pain due to rehabilitation, and stroke recurrence; however, no adverse events were observed in the present study. Only one patient with PG dropped out due to a lack of motivation. Table 1 shows the characteristics of patients in the PG and CG. There were no significant differences at baseline in these parameters between the two groups (all *P* > 0.196). The ratio of female to male patients (PG, 5/3; CG, 3/5; *P* = 0.317) and the types of disease (haemorrhage/infarction) were not different between the groups: 3/4 in both groups (*P* = 1.000). The latency between the time of onset and the study was 42 (33) and 66 (38) months in the PG and CG, respectively (*P* = 0.197). The data of patients at the time of enrollment were not significantly different. The authors failed to obtain FMA data only in one patient in the PG, since the patient refused to provide it, and there was no difference between the groups (*P* = 0.828).

**Table 1:** Characteristics of the participants

	PG (n = 8)	CG (n = 8)	*P* values
Age, years	76.1 (5.3)	75.6 (6.9)	0.873
Sex, women/men	5/3	3/5	0.317
Height, cm	153.4 (4.5)	157.4 (8.9)	0.269
BMI, kg/m^2^	23.11 (1.68)	23.10 (3.10)	0.993
Disease type, haemorrhage/infarction	3/5	3/5	1.000
Paralyzed side, right/left	4/4	3/5	0.614
Use of walking brace	1	1	1.000
Use of cane	4	4	1.000
Time since onset, months	42 (33)	66 (38)	0.196
Fugl-Meyer Assessment (total 226) ¶	210.7 (10.8)	209.3 (14.3)	0.828
Motor function score of FMA (total 100) ¶	90 (5.5)	92.4 (3.6)	0.854

Values are shown as means (SD) and numbers of each relevant item for a total of 16 ambulatory patients with chronic cerebrovascular disease. PG, intervention group, in which participants ingested high protein jelly within 30 min after each exercise session (n = 8; containing 10 g protein); CG, control group, intaking protein-free jelly (n = 8); Fugl-Meyer Assessment (FMA) is a stroke-specific, performance-based impairment index [28]. FMA, an index of physical dysfunction in patients with hemiplegia, consisted of assessments for motor functions of the upper (66) and lower extremities (34), sensory (24), balance (14), ranges of motion (44), and joint pain during exercise (44), with a total score of 226 in 7 and 8 participants in PG and CG, respectively ¶. Regarding motor function score of FMA, which consists of upper and lower extremities, total of which is 100, Duncan et al. classified as follows: 0–35, severe; 36–55, moderately severe; 56–79, moderate; and 80 or greater as mild [53]. P values of the unpaired t-test or χ^2^ test are shown

The average Borg’s scale score during intervention was approximately 12 in each group, with no significant differences between the groups (*P* > 0.783; Fig. 3).

The calorie intake from meals per day, except for the jelly intake just after exercise, were 26.0 (5.9) kcal/kg BM/day and 26.0 (6.5) kcal/kg BM/day of total energy, 1.12 (0.27) g/kg BM and 1.09 (0.23) g/kg BM of proteins, 3.73 (0.84) g/kg BM and 3.69 (0.89) g/kg BM of carbohydrates, 0.70 (0.21) g/kg BM and 0.74 (0.22) g/kg BM of lipids, and 0.11 (0.02) g/kg BM and 0.10 (0.03) g/kg BM of salt in the PG and CG, respectively, with no significant differences between the two groups (all, *P* > 0.687). (Table 2).

**Table 2:** Diet during intervention

	PG (n = 8)	CG (n = 8)	*P* values
Total energy, kcal/kg BM/day	26.01 (5.89)	26.01 (6.47)	0.999
Proteins, g/kg BM/day	1.13 (0.27)	1.09 (0.23)	0.749
Carbohydrates, g/kg BM/day	3.73 (0.84)	3.69 (0.89)	0.935
Lipids, g/kg BM/day	0.70 (0.21)	0.74 (0.22)	0.687
Salt, mg/kg BM/day	108.40 (24.35)	103.31 (26.47)	0.695

The diet was provided every day for all patients. Values are shown as the mean (SD)

*P* values are for the unpaired t-test

As shown in Table 3, there were no significant medication biases. Table 4 shows the [Hb], Ht, blood sugar, and serum profiles before the intervention; there were no significant differences between the two groups (all *P* > 0.170).

**Table 3:** Medication content in both groups

Types of medicines	PG (n = 8)	CG (n = 8)	*P* values
Antihypertensives			
Calcium blocker	3	5	0.317
α blocker	0	1	0.302
β blocker	1	1	1.000
ARB	3	3	1.000
Diuretics	2	1	0.522
Antithromboembolism			
Anticoagulants	2	1	0.522
Antiplatelet agents	5	6	0.590
Antithyperlipidemia (HMG-CoA reductase inhibitor)			
	5	5	﻿1.000
Antidiabetic drug			
	2	0	0.131
Osteoporosis drug (an analogue of vitamin D)			
	5	4	0.614
Analgesics			
NSAIDs	0	2	0.131
Pregabalin	2	2	1.000
Antidepressant			
SNRI	0	1	0.302
SSRI	1	1	1.000
Nonbenzodiazepine	1	1	1.000
Benzodiazepine	1	3	0.248
Gastric secretion inhibitor/ mucosal defense			
Proton pump inhibitor	6	3	0.131
H_2_ receptor antagonist	0	2	0.131
Gastric mucosal defense	0	3	0.055
Cardiorespiratory disease drugs			
Bronchodilator (β agonist)	0	1	0.302
Antitussive	0	1	0.302
Anti-allergic (antihistamine)	1	0	0.302
Digitalis	1	0	0.302
Urologic disease drugs			
Anticholinergic agent	1	0	0.302
β_3_ agonist	0	1	0.302
Urinary tract antispasmodic	0	1	0.302
Others			
Laxative	3	3	1.000
Thyroid hormone preparation	1	0	0.302
Antihyperuricemic	1	1	1.000
Acetylcholinesterase inhibitor	1	1	1.000

The numbers of medications in the intervention (PG) and control groups (CG) are shown

ARB, angiotensin II receptor blocker; HMG-CoA, 3-hydroxy-3-methylglutaryl-coenzyme A; NSAIDs, non-steroidal anti-inflammatory drug; SNRI, serotonin-noradrenaline reuptake inhibitor; SSRI, selective serotonin reuptake inhibitor

The *P* values of the χ^2^ test were described

**Table 4:** Haemoglobin concentration, haematocrit, blood sugar, and serum profiles before and after the intervention

Measurements	PG (n = 8)		CG (n = 8)		*P* values
	Before	After	Before	After	Time Group Group × Time
[Hb], g/dL	12.6 (1.9)	12.7 (2.2)	13.0 (1.7)	12.9 (1.9)	0.781 0.753 0.734
Haematocrit, %	37.3 (5.6)	37.9 (5.2)	38.9 (4.5)	39.0 (4.7)	0.415 0.584 0.582
[Albumin]_s_, g/dL	4.1 (0.4)	4.0 (0.2)	4.4 (0.2)	4.2 (0.3)	0.133 0.136 0.862
[Creatinine]_s_, mg/dL	0.85 (0.27)		0.87 (0.21)		0.888
C-reactive protein, mg/dL	0.1 (0.2)		0.3 (0.2)		0.204
Blood sugar, mg/dL	121.8 (23.5)		119.0 (21.0)		0.809
[Total cholesterol]_s_, mg/dL	184.0 (32.8)		188.0 (25.7)		0.790
[Triglycerides]_s_, mg/dL	210.1 (77.0)		204.0 (132.0)		0.911

Data are median values (SD) in the intervention (PG) and control groups (CG)

[Hb], haemoglobin concentration; []_s_, serum concentration of each relevant item. Ht, haematocrit, and albumin levels were assessed by two-way repeated measures ANOVA

*P* values of the effects of group, time, and interaction group and time are shown. The *P* values of the other samples compared by the unpaired t-test are also shown

**Fig. 5:**
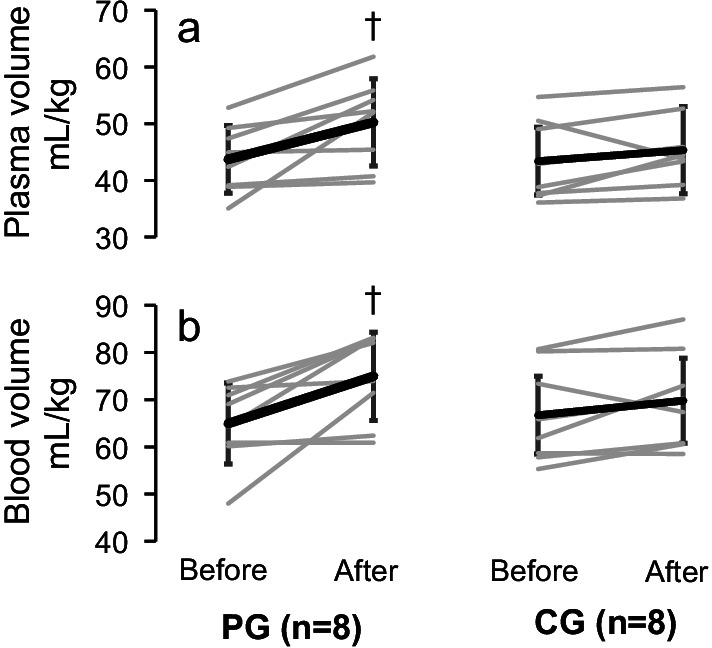
Plasma (PV; panel **a**) and blood volumes (BV; panel **b**) before (before) and after the intervention (after) in the intervention (PG; n = 8) and the control groups (CG; n = 8). PG in left, CG in right. Values are shown as means ± SD. †, significant differences from “Before” at the level of *P* < 0.05

As shown in Fig. 5, PV and BV increased after the intervention in the PG (*P* = 0.005 and *P* = 0.002 of time effect of Two-way ANOVA, respectively), while it remained unchanged in the CG. The 95% confidence intervals for PV were [1.5, 11.6] and [− 1.4, 5.3] and for BV were [2.8, 17.2] and [− 1.3, 7.7] in the PG and CG, respectively.

There were no significant differences in baseline physical fitness variables. Body weight decreased after the intervention in both groups (*P* = 0.004). Four of the patients in the PG, five in the CG, and one of the patients who performed both trials, took antihypertensive medication. Blood pressure in patients did not increase after the intervention, and no additional antihypertensive medications were used during their admission. Diastolic pressure tended to decrease only in the CG (*P* = 0.059). The mean arterial pressure and rate-pressure product decreased only in the CG (*P* = 0.049 and 0.047, respectively). The 6MWD increased only in the PG, with a significant interaction between group and time (*P* = 0.037). Confidential intervals of 95% were [26, 83] and [− 12.7, 43.2] in the PG and CG, respectively. VO_2peak_ and the strength of knee extension in the paralysed limb increased in both groups (*P* = 0.035 and *P* = 0.001, respectively). The 10MWS at optimal speed increased significantly in the PG (*P* = 0.019); however, there was no significant interaction between group and time (*P* = 0.597). The 10MWS at maximal speed remained unchanged in both groups (*P* = 0.116; Table 5).

**Table 5:** Body weight, cardiovascular responses, physical fitness, and walking performance before and after 3-week rehabilitation in both groups

Measurements	PG (n = 8)		CG (n = 8)		*P* values
	Before	After	Before	After	Time Group Group × Time
Body weight, kg	54.5 (6.5)	52.9 (6.2)†	57.5 (10.8)	55.4 (9.5)†	0.004 0.532 0.639
Systolic blood pressure, mmHg	119.8 (17.6)	121.9 (17.6)	128.3 (11.3)	120.9 (10.8)	0.401 0.584 0.139
Diastolic blood pressure, mmHg	82.0 (12.2)	81.1 (15.3)	88.5 (7.9)	76.3 (11.5)	0.046 0.878 0.079
Pulse pressure, mmHg	37.8 (9.4)	40.8 (12.7)	39.8 (10.0)	44.6 (13.7)	0.274 0.538 0.790
Mean arterial pressure, mmHg	94.6 (13.5)	94.7 (15.0)	101.8 (7.8)	91.1 (9.2) †	0.052 0.737 0.052
Rate-pressure product	8713 (1780)	8864 (1781)	9749 (1583)	8798 (1519) †	0.129 0.553 0.043
Heart rate, beats/min					
Rest	72.6 (8.6)	72.8 (10.0)	76.6 (14.4)	72.8 (10.0)	0.307 0.706 0.277
Peak	125 (21)	127 (19)	127 (18)	125 (19) ¶	0.976 0.978 0.694
VO_2_, mL/kg/min					
Rest	4.8 (1.2)	4.4 (0.9)	5.9 (1.3)	5.2 (1.3)	0.049 0.109 0.514
Peak	17.7 (3.9)	19.3 (2.2)†	19.6 (4.0)	21.2 (4.4)†	0.035 0.276 0.994
VO_2_, mL/min					
Peak	956.1 (211.1)	1022.0 (182.0)	1134.8 (335.8)	1187.7 (340.2)	0.136 0.217 0.866
Knee-extensor strength, Nm					
Paralyzed	35.9 (13.7)	39.6 (13.8)†	33.6 (6.7)	36.8 (5.7)†	0.001 0.636 0.798
Non-paralyzed	48.2 (10.2)	48.9 (10.6)	43.9 (8.0)	44.2 (6.2)	0.532 0.322 0.826
6MWD, m	288 (106)	342 (108)†	281 (110)	296 (117)	0.001 0.641 0.037
10MWS, m/sec					
Optimum	0.84 (0.32)	0.95 (0.31)†	0.78 (0.25)	0.85 (0.33)	0.019 0.597 0.530
Maximum	1.12 (0.42)	1.26 (0.40)	1.08 (0.49)	1.09 (0.55)	0.116 0.116 0.181

Values are shown as mean (SD) in the intervention (PG) and control groups (CG). Systolic (SBP) and diastolic blood pressure (DBP) were measured at rest before cardiopulmonary exercise testing. The heart rate at peak was recorded during cardiopulmonary exercise testing in 8 and 7 participants (¶) in PG and CG, respectively. The pulse pressure was calculated as SBP – DBP. The mean arterial pressure was calculated as DBP + (SBP − DBP)/3. The rate-pressure product was calculated as the heart rate × SBP. VO_2_, oxygen consumption rate; 6MWD, 6-min walking distance; 10MWS, 10-m walking speed; †, compared with the baseline before the onset of rehabilitation program at the level of *P* < 0.05, as determined by the paired t-test

*P* values of the effects of group and time and interaction group and time from two-way ANOVA repeated measurement are shown

Table 6 shows that concentrations of cortisol and catecholamines remained unchanged before and after the intervention in both groups (all *P* > 0.134). However, plasma concentration of aldosterone decreased only in the PG (*P* = 0.028).

**Table 6:** Effects of 3-week rehabilitation and supplements on plasma concentrations of cortisol, catecholamines and aldosterone

	PG (n = 8)		CG (n = 8)		*P* values
	Before	After	Before	After	Time Group Group × Time
[Cortisol]_p_, μg/dL	12.27 (4.84)	13.02 (2.95)	10.30 (2.58)	10.37 (1.79)	0.581 0.132 0.674
[Adrenaline]_p_, pg/mL	26.75 (13.22)	28.25 (11.91)	36.13 (17.30)	38.50 (25.02)	0.479 0.262 0.872
[Noradrenaline]_p_, pg/mL	386.38 (259.28)	460.63 (273.52)	512.25 (363.39)	560.38 (342.37)	0.134 0.469 0.739
[Dopamine]_p_, pg/mL	8.50 (4.07)	12.75 (5.20)	16.63 (9.80)	16.63 (7.69)	0.264 0.067 0.264
[Aldosterone]_p_, pg/mL^a^	78.7 (38.4)	60.2 (24.5)†	47.98 (33.1)	46.25 (17.63)	0.046 0.204 0.089

Plasma concentrations of cortisol ([cortisol]_p_), adrenaline ([adrenaline]_p_), noradrenaline ([noradrenaline]_p_), dopamine ([dopamine]_p_), and aldosterone ([aldosterone]_p_) are shown as means (SD) in the intervention (PG) and the control group (CG)

*P* values of the effects of group and time and interaction group and time from two-way ANOVA repeated measurement are shown. †, compared between before and after the onset of rehabilitation program at the level of *P* < 0.05

^a^n = 6 because of the lack of samples

## Discussion

This is the first study in patients with chronic cerebrovascular disease that examined whether PV and BV increased with rehabilitation, along with improved physical endurance. The regimen of protein intake immediately after each exercise session in the 3-week rehabilitation program was used in the present study. As a result, PV and BV increased only in the PG, with an interaction of group and time, along with increased 6MWD and decreased plasma aldosterone concentration after the intervention. However, VO_2peak_ and strength of knee extension muscles in the paralysed limb increased regardless of protein intake. Although BV increased in the PG, blood pressure did not increase in patients with no change in plasma concentrations of stress-related hormones or catecholamines.

The 6MWD, as an index of physical endurance, improved only in the PG and was related to increased BV and PV. As previously reported, a 3-week period was sufficient to improve respiratory function and blood flow to the active muscles in the present patients [7, 8, 29–31]. Femoral arterial blood flow in the paralysed limb increased after a 2-week training intervention in post-stroke patients [31]. The increase in BV contributes to increased blood flow due to an increase in venous return to the heart [32]. The expansion of BV in the PG was induced by the following regimen: 3-week rehabilitation plus protein ingestion just after each exercise bout, as previously shown in healthy elderly [16] and hypertensive patients [14]. Thus, the present study shows that increased BV and/or PV may result in improved the 6MWD in patients with chronic cerebrovascular disease.

The reduction in plasma aldosterone concentration only in the PG after the intervention may have been due to increased PV and BV. As aldosterone release typically increases after exercise at moderate or high intensities [33, 34], this hormone increased after each rehabilitation session. In the present study, stimulation by aldosterone may have contributed to fluid volume expansion, while a decrease in the baseline level could not be explained. Fluid expansion observed only in the PG seemed to decrease it after the intervention.

Previous studies have described an increase in the rate of liver albumin synthesis following a single bout of intensive exercise [35]. Insulin suppresses proteolysis in the liver and enhances albumin synthesis [36]. Generally, one g of albumin draws 18 [37] or 14–15 mL of water [38]. The increase in PV is associated with an increased gradient of colloid osmotic pressure [38]. Protein synthesis after exercise in groups with a high (1.8 g/kg/day) and low (0.7 g/kg/day) protein intake the day before the exercise session was comparable in young subjects [39]. Protein intake (0.18 g/kg) 2 h after a bout of high-intensity interval exercise did not result in a change in PV 24 h after the exercise; however, ingestion of the same amount of protein just after the exercise resulted in increased PV [40]. Thus, protein intake immediately after exercise would be more beneficial for expanding PV. In the present study, PV increased only in the PG, while [Albumin]_s_ remained unchanged after the intervention, suggesting that albumin content in plasma may increase only in the PG. The reason why PV and BV significantly increased only in the PG is explained by the greater gradient of the colloid osmotic pressure, and the timing of the ingestion worked effectively.

VO_2peak_ and muscle strength in the paralysed limb improved during the 3-week rehabilitation program in both groups. As patients in the present study were not physically active before the study, they had a high potential for trainability, including one patient who continued rehabilitation therapy twice a week in the PG. The discrepancy between improvements in VO_2peak_ and 6MWD in both groups remains unclear. However, it involves factors other than PV and/or BV.

Since anaemia sometimes disturbs the progress of increasing workload and/or physical activity during rehabilitation [41–43], it should be corrected immediately. In this study, two patients in the PG and three patients in the CG were classified as anaemic before the intervention based on the World Health Organization criteria [18]: < 13 and 12 g/dL for men and women, respectively. These patients remained anaemic after the intervention. However, PV also increased in all patients in the PG and some patients in the CG, while [Hb] levels decreased in the patients. [Hb] and haematocrit remained unchanged after the present intervention, and haemoglobin content also increased in the PG. Previous studies showed that a 2-week endurance training in healthy subjects increased plasma concentrations of erythropoietin, a haematopoietic factor secreted by tubular stromal cells in the kidney, which is known to promote erythrocyte production [44]. Protein intake immediately after exercise can also increase haemoglobin content, possibly through enhanced production of haematopoietic hormones.

The patients in the current study performed several resistance exercises, such as squats and calf raise exercises, as well as walking. Improvement in muscle strength in the paralysed limb was achieved after just 3 weeks. Three weeks is generally too short to induce muscle hypertrophy in healthy persons [45, 46]; indeed, at least 6 weeks are needed [47]. Therefore, there was no detectable limb hypertrophy after the 3-week period in the non-paralysed limb. Improvements in neuromuscular recruitment in the paralysed muscles, learning of motor timing and sequencing for locomotion, and coordination of agonist/antagonist muscles are factors that may be responsible for increased muscle strength [48–50]. Indeed, approximately 20% of the corticospinal tract fibres do not cross at the medulla and descend on the ipsilateral side as part of the anterior corticospinal tract [51]. Rehabilitation may increase the activity of the tract.

We could not find any reasonable explanation for improvements in 10MWS at the optimal speed only in the PG. Habitual gait speed correlates with VO_2peak_ [9] and maximum gait speed correlates with paretic knee extension muscle strength [52]. In the present study, 6MWD improved only in the PG, suggesting an enhancement of physical endurance. The improvements in 10MWS in the PG could not be explained by muscle strength in the lower limbs; however, improved range of motion of the ankle may partially influence improvement of 6MWD.

An expansion of BV is believed to be a risk factor for increased blood pressure. However, there were no cases in which blood pressure at rest was elevated after the intervention, and no medications were altered during the admission in the present study. Baseline plasma concentrations of catecholamine and stress-related hormones remained unchanged after the present intervention, suggesting that sympathetic nervous activity would not be enhanced, even after BV expansion. This is due to the fact that blood pressure regulation via baroreflexes can be improved by enhancing the reflex responses [14].

### Limitations

This was a single-site study, and the sample size was limited. However, the preliminary results of the present study may help support planning of the next multiple-centre study.

## Conclusions

Rehabilitation therapy for 3 weeks combined with protein intake immediately after each exercise session increased PV and BV in patients with chronic cerebrovascular disease. Physical endurance, especially 6MWD, improved with protein intake. Furthermore, blood pressure did not deteriorate in the present study, even when BV increased after the intervention.

## Supplementary Information

**Additional file 1:** Individual data of Figures 3 and 5 and all Tables are shown in the additional file, while data of "Sex" and "Age" are masked.

## Data Availability

The datasets used and/or analysed during the current study are available from the corresponding author on reasonable request. The data except for some of indirect identifiers are also available in the Additional file 1: Data.

## Abbreviations

BV: Blood volume
CG: Control group
CV: Coefficients of variation
FMA: The Fugl-Meyer Assessment
[Hb]: Haemoglobin concentration
PG: Intervention group (ingested protein immediately after each exercise session)
PV: Plasma volume
VO_2peak_: Peak oxygen consumption rate
6MWD: 6-min walk distance at optimal speed
10MWS: 10-M waking speed at optimal and maximal effort
